# The Role of Adipokines in Surgical Procedures Requiring Both Liver Regeneration and Vascular Occlusion

**DOI:** 10.3390/ijms19113395

**Published:** 2018-10-30

**Authors:** Ana Isabel Álvarez-Mercado, Esther Bujaldon, Jordi Gracia-Sancho, Carmen Peralta

**Affiliations:** 1 Experimental Liver Surgery and Liver Transplantation, Institut d’Investigacions Biomèdiques August Pi I Sunyer (IDIBAPS), 08036 Barcelona, Spain; analvarezmercado@gmail.com (A.I.A.-M.); ebujaldonormaechea@gmail.com (E.B.); 2 Centro de Investigación Biomédica en Red de Enfermedades Hepáticas (CIBEREHD), 28029 Madrid, Spain; jordi.gracia@idibaps.org; 3 Liver Vascular Biology Research Group, IDIBAPS, 08036 Barcelona, Spain; 4 Facultad de Medicina, Universidad Internacional de Cataluña, 08017 Barcelona, Spain

**Keywords:** ischemic/reperfusion injury, liver regeneration, adipokines, partial hepatectomy

## Abstract

Liver regeneration is a perfectly calibrated mechanism crucial to increase mass recovery of small size grafts from living donor liver transplantation, as well as in other surgical procedures including hepatic resections and liver transplantation from cadaveric donors. Regeneration involves multiple events and pathways in which several adipokines contribute to their orchestration and drive hepatocytes to proliferate. In addition, ischemia-reperfusion injury is a critical factor in hepatic resection and liver transplantation associated with liver failure or graft dysfunction post-surgery. This review aims to summarize the existing knowledge in the role of adipokines in surgical procedures requiring both liver regeneration and vascular occlusion, which increases ischemia-reperfusion injury and regenerative failure. We expose and discuss results in small-for-size liver transplantation and hepatic resections from animal studies focused on the modulation of the main adipokines associated with liver diseases and/or regeneration published in the last five years and analyze future perspectives and their applicability as potential targets to decrease ischemia-reperfusion injury and improve regeneration highlighting marginal states such as steatosis. In our view, adipokines means a promising approach to translate to the bedside to improve the recovery of patients subjected to partial hepatectomy and to increase the availability of organs for transplantation.

## 1. Introduction

The ability of the liver to regenerate even when 70% of the organ tissue has been removed [1] together with the shortage of liver grafts from deceased donors have led to an increased interest in living donor liver transplantation (LDLT), where healthy donors undergo anatomical hepatectomy [2], and split liver transplantation from cadaveric donors to be used in two recipients [3]. Both procedures require liver regeneration [4] and are associated with inherent process of cold ischemia, which negatively affect post-operative outcomes [5]. In addition, an important cause and unavoidable consequence of liver damage during partial hepatectomy (PH) associated with tumor hepatic resection is ischemia reperfusion (I/R) injury. I/R accentuates cellular damage [6], significantly reduces liver regeneration, and induces apoptosis and necrosis in hepatocytes [7,8].

On the other hand, there is a close crosstalk between the adipose tissue and the liver. Substances produced at the visceral adipose tissue level directly target the liver through the portal vein [9]. In hepatic resections, the presence of fatty infiltration is associated with poor outcome after surgery and pre-existing steatosis is related with impairment of liver regeneration following PH [4]. Moreover, expansion of white adipose tissue is associated with both pathological or, on the contrary, with protective and regenerative conditions [10].

Adipokines are signaling and mediator proteins secreted mainly but not exclusively by adipose tissue [11]. Indeed, a considerable amount of adipokines is synthesized in the liver (i.e., leptin, adiponectin, retinol binding protein 4 (RBP4), chemerin, angiotensinogen, delta like-1 homologue, insulin growth factor, hepatocyte growth factor, lipocalin2 (LCN2), etc.). Most of them are related either with the promotion or reduction of regeneration, although some studies had also reported effects in liver function and liver damage [10,12,13,14,15,16,17,18,19,20,21,22,23,24,25,26,27,28,29,30,31,32,33,34]. Besides, the secretion of adipokines may contribute to the development of metabolic diseases including fibrosis and cirrhosis [11]. Conversely, certain adipokines may have anti-inflammatory and anti-obesity properties [35]. Altogether this emphasizes the importance of further characterizing the adipocyte secretion profile and the role of adipokines during liver regeneration. Therefore, the understanding of such underlying mechanisms and those that ameliorate livers from I/R injury would provide novel therapeutic approaches to be transferred to clinical conditions and consequently increase the number of available donors for transplantation and improve recovery for patients subjected to PH.

Table 1 summarizes the main adipokines involved in liver diseases and/or regeneration. Accordingly, in the present review, we discuss the results published in the last five years based on such adipocytokines in different surgical procedures (small-for-size liver transplantation and hepatic resections) requiring both regeneration and vascular occlusion (which increase I/R injury) with special emphasis in marginal liver status such as steatosis.

## 2. Partial Hepatic Resection

Hepatic resection in rodents is frequently performed to study liver regeneration and liver responses to stress [36]. After partial resection, liver regeneration is a perfectly calibrated response whose apparent sensor is the requirement of the body for liver function. Many genes involved in the generation of a cytokine network are differentially expressed during the first few hours after PH, considered the “priming phase” in regeneration [37]. A considerable amount of them are specifically adipokines. The main changes in cytokines expression associated to the mechanism of liver regeneration under hepatic resection conditions are as follows: After PH hepatocytes are primed by induction TNF-α and IL-6 in Kupffer cells resulting in activation of STAT3. Afterwards, the increased expression of TGFα, HGF, and EGF induces proliferation and hepatocytes growth [38]. HGF stimulation (defined as a major regulator of hepatocyte proliferation) is mediated by stellate cells as well as endothelial cells via VEGF receptor achieving the peak of mRNA expression in liver 12 h after PH, when most of the hepatocytes are already in S-phase. In addition, HB-EGF seems to play a distinctive role in liver regeneration after PH and is expressed even earlier than HGF and TGF [39].

Adipokines also play important roles in I/R injury caused during PH surgery as well as in others surgical procedures [40,41,42,43,44]. 

Considering that the activation of numerous adipokines play a role in liver diseases and further evidences show their importance for regeneration (see Table 1), studies looking for specific functions and mechanisms of adipokines in liver regeneration and I/R injury during surgery, as well as strategies to improve regeneration and liver function recovery, are highly necessary.

From our knowledge, the study of adipokines in humans has mainly performed from serum samples without pharmacological modulation of adipokine actions.

In LDLT serum, adipokines have mainly been reported as biochemical markers to evaluate the risk of fibrosis progression in patients transplanted for hepatitis C [45] as well as in the diagnosis of non-alcoholic fatty liver disease (NAFLD) or non-alcoholic steatohepatitis (NASH) [46]. In healthy humans, Matsumoto et al. evaluated changes in levels of adipokines in the serum of patients subjected to PH, but they did not reported results related to the modulation of them. In this sense we found other study evaluating cytokine profiles related to regeneration in donors and recipients before and after LDLT but once again author did not modulate adipokine actions [47].

On the other hand, liver regeneration has been studied in patients undergoing liver resection with underlying diseases like some infectious diseases of the liver [48], surgical resection of liver cancers as well as in recipients of liver transplantation who usually suffer other diseases and immunosuppressant treatment after transplantation [38]. Also, the consideration of steatosis and other marginal liver status [49] in these models are strongly necessaries. In consequence, the profile of adipokines and the regulation of regeneration might differ and involve different signal transduction pathways depending on the conditions mentioned-above in both donors and recipients [47]. Therefore, all these variables need to be considered in animal models (vascular occlusion or not, underlying diseases, cancer, presence of steatosis or not, etc.) to precisely decipher how adipokines modulation may affect regeneration and liver function specifically in any condition.

Additionally, this remarkable ability to regenerate its mass after PH injury does not only involve proliferation but also hepatocytes compensatory hypertrophy and hyperplasia. In fact, it has been reported that mice cellular hypertrophy makes the first contribution to liver mass restoration. Additionally, in mice, regeneration after 30% of liver mass removal was achieved only by hypertrophy without cell division, whilst after 70% hepatectomy hypertrophy anteceded proliferation [50]. Accordingly, studies evaluating the specific role of adipokines in such mechanisms and their affectation by pathological states such as steatosis are mandatory.

Table 2 summarizes the reported literature evaluating the roles and mechanisms of adipokines in liver regeneration and damage in experimental models of PH with or without vascular occlusion and small-for-size liver transplantation (SFSLT) published in the last five years.

## 3. The Animal Models of PH

The model described by Higgins and Anderson is the most well known and extended experimental model of PH. In it, a compensatory hyperplasia is produced after removal of two-thirds (approx. 70%) of the organ by enlargement of the remaining lobes [36].

Most studies included in Table 2 were performed in the 70% PH models described either by Higgins and Anderson [36] or the similar by Mitchell and Willenbring [1]. Two of them [54,58] applied PH with the presence of I/R. Interestingly, only two studies have reported data approaching the effects of the modulated adipokine in experimental models of cancer [52,71]. Koh and colleagues found that the treatment with an inhibitor of the renin-angiotensin (Captopril) regresses colorectal cancer liver metastases induction after 70% PH without vascular occlusion without impairing liver recovery [71]. On the other hand, a work recently published by Lanton et al. using a model of chronic inflammation-associated liver cancer revealed that the inhibition of interleukin 6 (IL-6) signaling impeded tumorigenesis following PH without vascular occlusion and did not affect survival or recovery of the liver mass [52]. These are remarkable findings with clinical interest since these drugs regulating Ang II or IL-6 avoided cancer cells growth under PH. Under these conditions (the presence of hepatic tumors), IL-6 did not affect the recovery of the liver mass. On the contrary, results from PH experimental models (in the absence of tumorigenesis) indicate a relevant role of IL-6 on damage and liver regeneration [55,56]. Consequently, further studies will be required to elucidate the role of IL-6 in surgical conditions that resemble as much as possible the clinical surgical conditions, such as PH, vascular occlusion, the presence of hepatic steatosis, and tumorigenesis. 

### 3.1. Gene-Specific Null Mutations (KnockoutModels)

Since human adipokines have not yet been completely characterized (i.e., there are more than 600 potential adipokines unidentified) [83], several studies have been reported to selectively examine the precise function of every single adipokine through KO models. Indeed, many authors used knockout (KO) mice in the last five years, using 70% PH without vascular occlusion [23,24,52,64,66,73]. However, no one of the studies mentioned has evaluated the gene-specific null mutations of adipokines in steatotic livers, which represents a limitation to transfer to the bedside. In the pre-clinical scenario of KO models, controversial results on the role of adipocytokines in liver regeneration after PH have also been reported. For instance, in the case of an LCN2KO, after PH, this adipokine was massively induced in mice, although the authors did not find differences in regeneration when comparing with wild-type animals [24]. On the contrary, Xu et al. reported that hepatocyte-derived LCN2 after treatment with IL-6 promoted liver regeneration after PH. These discrepancies might be explained because the different methods to quantify liver regeneration [23]. However, it is worth noting the potential of this protein, a cytokine firstly used as a biomarker for renal injury and inflammation that later was described as the major positive acute-phase protein in rat during acute-phase reaction by Sultan et al. Indeed, they also found that the liver and not the kidney is the main source of serum LCN2 in the case of tissue damage [84]. This is a clear indication that we need further research about the role of adipokines in KO models under specific conditions in liver surgery requiring vascular occlusion and in the presence of fatty infiltration.

### 3.2. Steatotic Livers

These livers present affectation in the regenerative response and less tolerance to damage in comparison with non-steatotic livers [85,86,87]. In addition, numerous studies recently performed point to an important role for adipokines in hepatic steatosis. Both leptin and adiponectin play key roles in obesity-related disorders and have been associated with the pathogenesis of non-alcoholic fatty liver disease [88]. Published studies were not performed in the clinical setting of hepatic regeneration, but circulating levels were measured in NAFLD patients. In addition, in vitro studies have found a deep relation between leptin and liver fibrosis, showing a pro-fibrogenic role for this adipokine [89].

From our knowledge, only three studies characterizing adipokines in PH in steatotic livers have been published during the past five years [58,69,75]. Two of them reported results comparing steatotic and non-steatotic grafts under I/R conditions.

Elias et al. found increased visfatin production in PH under I/R. They also showed that steatotic livers were more vulnerable to upregulate visfatin than were non-steatotic livers. In addition, in steatotic livers following PH under I/R, the treatment with resistin modulated the detrimental effects on hepatic damage and regenerative failure induced by adipose tissue-derived visfatin [58].

Accordingly, Gu et al. found that steatosis hardly decreases survival and regeneration after expansive liver resection and this effect can be partly counteracted by perioperative treatment with VEGF [75] in PH without vascular occlusion conditions.

The third study published in this sense reported that, in mice, under PH+IR conditions, the overexpression of LCN2 produced a significant increase in hepatic damage associated to surgery while KO mice did not present this affectation [69].

### 3.3. Relevance of Vascular Occlusion under Partial Hepatectomy

To control bleeding in the course of parenchyma dissection, surgeons usually perform hepatic resection under vascular occlusion [90]. However, this implies hepatic I/R injury if surgeons clamp the liver vasculature for a long period of time [91]. To resemble as much as possible the clinical situation and understand the mechanisms in which adipokines are involved, experimental models including hepatic regeneration plus I/R injury are advisable. 

As mentioned above, in the last five years, studies involving adipokines in animal models of PH have mainly been performed with the removal of 70% of total liver mass, but only three of them added total vascular occlusion in the surgery [54,58,69] and only two compared liver with and without steatosis [58,69]. In addition, the extended period of I/R was very different (10–60 min) as well as its induction. Moreover, the presence of hepatic tumor has been only considered in two experimental studies of liver surgery without vascular occlusion and in non-steatotic livers [52,71].

However, it is important to note that results and signaling pathways on the role of adipokines and its regulation might be different when comparing different I/R times as well as studies performed with or without vascular occlusion. Additionally, steatotic livers present dysfunction in the regenerative response and decreased tolerance to I/R injury when compared with non-steatotic livers in the setting of PH with vascular occlusion [12,85,92]. Thus, the types of surgical procedures (PH performed with or without vascular occlusion) and liver status previous to surgery (steatotic or non-steatotic, the presence or absence of tumorigenesis, and the age of the animal) should be considered in preclinical studies since it may ultimately dictate the hepatocellular response and consequently the protective strategies required to reduce I/R damage and improve liver regeneration in liver surgery.

## 4. Animals Models of Partial Liver Transplantation

LDLT has become an important alternative to liver transplantation from deceased donors to solve the increasing problem of organ shortage. The use of small-for-size (SFS) grafts for LDLT present certain benefits due to the liver procurement from the living donor can be selectively timed with the recipient (i.e., this grafts present good quality and are exposed to short periods of ischemia) [90]. Nonetheless, regeneration which is inherent to the liver after certain surgical procedures and the mechanism of damage caused by the removal of hepatic mass and those associated with cold I/R should be considered when SFS are transplanted. Indeed, patients transplanted with SFS grafts appear to have a poorer prognosis after transplantation, and the regeneration of liver is markedly inhibited leading to compromised liver function and graft loss [4,77].

In liver resections with mass deletion of 40–70%, there is a linear relation between the % of tissue removed and the grade of proliferation of hepatocytes. However, resections larger than 70% of liver mass result in an increase in mortality [93]. Thus, it has been suggested that liver can meet the metabolic demands of the recipients with graft sizes of 40% or greater [94], and importantly this liver mass is able to tolerate the hyperperfusion occurring after LDLT using SFS grafts [95]. These observations should be considered in the design of experimental models of SFS grafts that resemble the clinical conditions. 

In the last five years, most reported results in SFSLT came from models either 50% or 30% transplanted liver mass and ischemia times from 10 min to 10 h. Although encouraging results related with the improvement of regeneration through the modulation of adipokines are shown, discrepancies in animal models and methodology seriously impair the applicability of results. Indeed, authors did not take into account factors such as the donor type or the use of ischemia time closer to the already used in clinical surgical procedures. In addition, further investigations will be required to elucidate the role of adipokines in split liver transplantation since the presence of brain death negatively affects liver damage and regenerative response [96]. Given these observations and the different times of cold ischemia (6–8 h in split liver transplantations versus 1 h in LDLT), the role of adipokines observed in LDLT might be not extrapolated to split liver transplantation.

### 4.1. Gene-Specific Null Mutations (KnockoutModels)

As part of an extensive and meritorious work where different surgeries including SFSLT where evaluated, Song et al. reported the use of IL-6 KO to evaluate changes in IL-6 after administration of melatonin concluding that the absence of IL-6 in these animals led to a failure in promoting liver mass recovery [55].

### 4.2. Steatotic Livers

In spite of the significant prevalence of hepatic steatosis in the society that would indicate how the outcome after SFSLT can be improved, we only found one study aimed at understanding the precise role of adipokines using this type of grafts in the last five years [68]. In it, the authors reported that upregulation of LCN2 promoted massive macrophage infiltration and exacerbated steatotic liver graft injury in a rat model of SFSLT. Regarding this study, it is noteworthy to remark the inclusion of a model of PH+IR also using fatty livers comparing LCN2^−/−^, wild type, and mice that overexpressed LCN2 (results mentioned above). Reported results contribute to clearly elucidate how modulation or inhibition of this adipokine affects in the different surgeries making possible to evaluate discrepancies in action mechanism depending on the type of surgery. However, in our view, the authors failed since they used different species (rat and mice) with make difficult to compare.

Based on the reported studies in the last five years, Table 3 summarizes future perspectives of the most relevant adipokines implied in regeneration and/or liver diseases as well as their applicability as potential new targets to decrease I/R injury and improve regeneration in PH and partial LT.

## 5. Concluding Remarks

**Figure 1:**
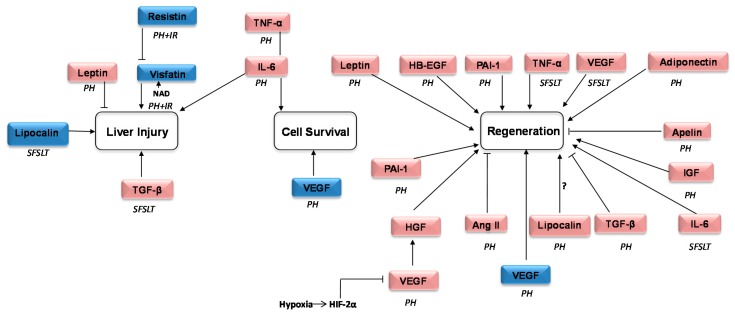
Schematic representation of the currently explored adipokines reported as involved in regeneration and/or liver injury in surgical procedures requiring liver regeneration and vascular occlusion in the last five years. Pink: adipokines in non-steatotic liver surgery. Blue: adipokines in steatotic liver surgery. The symbol ? means that controversial results have been reported. ↑ Promotion or activation of the process. ┤ Inhibition or decrease of the process. PH: partial hepatectomy; I/R: ischemic reperfusion; SFSLT: liver resection and small-for-size liver transplantation; HGF: hepatocyte growth factor; VEGF: vascular endothelial growth factor; TGF-β: tumor growth factor-β; HB-EGF: heparin binding: epidermal growth factor; PAI-1: phosphoribosylanthranilate isomerase 1; IL-6: interleukin 6; TNF-α: tumor necrosis factor; Ang: angiotensin; HB-EGF: heparin binding epidermal growth factor; NAD nicotinamide adenine dinucleotide; IGF: insulin growth factor; HIF-2 α: hypoxia-inducible factor alpha.

Adipokines participate in I/R injury and are related with inflammation, metabolic control, tissue repair, lipid metabolism, and certain liver disorders. These properties may warrant investigations using them as potential targets to counteract hepatic failure and improve regeneration after surgical interventions, like liver resection and liver transplantation. Considering this, it is key to resemble as much as possible at the bench-side the clinical situation during surgical procedures and to understand in detail the mechanisms in which adipokines are involved. Although important advances in this sense have been achieved in the last five years (Figure 1), it should also be considered that limitations of the preclinical studies performed, mainly in LDLT or split liver transplantation, may limit the transferability of their results.

Under this premise, several additional points need to be addressed before transferring to the clinic the current experimental knowledge.

First, in spite of being identified in models of regeneration and liver disease, we miss more results in PH models under I/R conditions. In addition, researchers need to decipher the precise roles played by individual adipokines since many of them are not clearly understood.

Other not less important issue is the large amount of surgical variables to take into account and the fact that, at less until now, there are no consensuses in their use and, therefore, methods and procedures for the same surgical procedure are not standardized (vascular occlusion or not, I/R time, age of the animal). Additionally, the real grade of the resection that may ultimately dictate the hepatocellular response should be considered. In the case of liver transplantation, it is necessary to consider the condition of the donor and/or the recipient such as subjacent pathologies or the need of immunosupressant treatment as well as the origin of the graft (LDLT or cadaveric). There are no data on how modulation of adipokines can affect their regeneration cascade and, in consequence, the clinical outcomes for recipients. Thus, these studies are mandatory given that they might alleviate the waiting list for transplant. Interestingly, we found two different studies evaluating different types of surgeries in the same work [54,69]. To some extent, findings from these may help explain discrepancies between regeneration and damage mechanisms depending on the method used.

In the case of marginal liver status as steatosis, it is very important to decipher how these livers respond to the modulation of adipokines given the fact that they follow different signaling pathways, and results, consequently, might be different than in non-steatotic livers. Similar considerations apply for aged versus young grafts.

On the other side of the coin, it is noteworthy that the effort of researchers to selectively examine the precise function of adipokines in KO models. However, the presence of both vascular occlusion and hepatic steatotis should be taken into account, since both conditions are of high prevalence in the liver surgery. Indeed, the most we know about the function of adipokines and pathways in which they are involved, the better in order to improve regeneration and liver function after surgery which will alleviate the waiting list for transplantation and recovery for patients requiring liver resection.

## Figures and Tables

**Table 1:** Prevalent action of some adipokines related to liver diseases and/or regeneration.

Role	Name	Prevalent Described Action
**Inflammation**	Chemerin	Mediates inflammatory responses, serving as a chemo attractant to induce influx of macrophages and natural killer cells [12]
	IL-6	Regulator of both the immune and the nervous system as well in liver regeneration [13]
	Omentin	Inhibitor of vascular endothelial cells inflammation. Related to heart vasculature disease and insulin sensitivity [14]
	PAI-1	Interacts with vascular cells. It has been related with angiogenesis and pro-inflammatory cytokines. Widely associated with insulin resistance and impaired immune response [15]
	Resistin	Involved in the pathogenesis of obesity, adipogenesis and insulin metabolism [16]
	TGF-β	Essential in establishing immunological tolerance. Pro-inflammatory roles in inflammatory responses [17]
	TNFα	As pro-inflammatory cytokine is involved in the development of many inflammatory diseases. “Master-regulator” of inflammatory [18] cytokines. Regulation of critical cell functions including cell proliferation, survival, differentiation, and apoptosis [10,18]
**Metabolic control**	Adiponectin	Involved in the pathogenesis of diabetes mellitus, obesity, hypertension, renal failure and atherosclerosis [19]
	Apelin	Takes part in the regulation of the physiology and pathophysiology of the circulatory system. Regulator of the metabolic balance, inflammation as well as cell proliferation and apoptosis [20]
	Leptin	Between other functions, regulates angiogenesis, hematopoiesis, carcinogenesis, satiety, energy expenditure and the immune system [21]
	Lipocalin	LCN2 in mainly produced by hepatocytes under acute-phase conditions. Considerable increased under stressed conditions like bacterial infection, surgical procedures or metabolic stress, plays an important role in suppressing bacterial infection by binding to bacterial catecholate-type ferric siderophores and consequent suppression of bacterial growth through the sequester of iron-laden siderophores [22]. LCN2 acts as immunomodulator and inhibitor of differentiation of erythroid progenitor cells and promotes apoptosis [23]
**Metabolic control**	RBP4	Retinol transportation in the circulation [24]
	Vaspin	Potential insulin-sensitizing effects [25]. Related with non-alcoholic fatty liver disease [26]
	Vifastin	Control of energy balance and insulin sensitivity. Regulates lipid metabolism and fatty acid oxidation [27]
**Regeneration**	Angiotensinogen	It is implied in the development of liver cirrhosis, portal hypertension, angiogenesis and apoptosis [28]
	Dlk-1	Adipogenesis, osteogenesis. Neuronal and neuroendocrine differentiation [29]
	HB-EGF	The soluble form induces mitogenic and regenerative activities [3]
	HGF	Proliferation, morphogenesis and anti-apoptosis [31]
	IGF	Both prenatal and postnatal development, including cell growth, differentiation, migration, and survival [32]
	NGF	Stimulation of growth, differentiation, survival and maintenance of neurons [33]
	VEGF	Regulator of angiogenesis also promotes collateral vessel growth [34]

IL-6: interleukin 6; TNF-α: tumor necrosis factor alpha; LCN: lipocalin; RBP4: retinol-binding protein 4; HB-EGF: heparin-binding epidermal growth factor; VEGF: vascular endothelial growth factor; TGF-β: tumor growth factor-β; PAI-1: phosphoribosylanthranilate isomerase 1; DKL-1: delta like-1 homologue; IGF: insulin growth factor; NGF: nerve growth factor; HGF: hepatocyte growth factor.

**Table 2:** Summarizes reported studies performed in partial hepatectomy (PH) models evaluating roles and mechanisms of adipokines in liver regeneration and damage in the last five years.

Name	Experimental Model			Effect on Liver Function and Regeneration	Reference
	Surgical Procedure		Specie		
	PH	PartialLT			
**IL-6**	68% PH		Mouse	In NO KO mice, impairment of IL-6 induction provoked excess of hepatic lipid accumulation, increased ER stress and negatively affected hepatocyte proliferation after surgery	[51]
	2/3 PH		Mouse	In multidrug resistance 2 knockout (Mdr2^−/−^) mice, pharmacological inhibition of IL-6 signaling inhibited tumorigenesis but did not affect survival or recovery of liver mass after PH	[52]
	78% PH		Mouse	A20 (an NF-κB inhibitory protein) promotes liver regeneration through enhance IL-6/STAT3 proliferative signals	[53]
	70% PH+I/R 1 h warm ischemia		Mouse	Melatonin protected from hepatic damage and promoted IL-6 and TNF-α and liver regeneration	[54]
	80% PH		Mouse	Melatonin-associated IL-6 increased liver microcirculation and survival	[54]
	70% PH		Rat	IL-6 regulated Mcl-1L (a member of the Bcl-2 family) expression through the JAK/PI3K/Akt/CREB signaling pathway. Mcl-1 inhibited apoptosis	[55]
		SFSLT (30%) 1 h cold ischemia	Mouse	Melatonin activated the IL6/GP130-STAT3 pathway protecting SFS graft and promoted regeneration	[54]
		SFSLT (30%) 1 h cold ischemia	Rat	The administration of Gadolinium chloride (GdCl3), a Kupffer cells inhibitor inhibited IL-6/p-STAT3 signal pathway, and thus in turn increased apoptosis and suppressed liver regeneration	[56]
**PAI-1**	70% PH		Mouse	Knocking out PAI-1 mice was associated with a decrease in hepatocyte proliferation	[57]
**Resistin**	70% PH+I/R 1 h warm ischemia		Rat: Steatotic and non-steatotic livers	Steatotic livers were more resistant to the overexpression of resistin after PH under I/R. Resisting originated in liver regulated the visfatin deleterious effects on inflammation and damage	[58]
**TGF-β**	2/3 PH		Mouse	Knockout of kupffel-like factor 10, an activator of the TGF-β/Smad signaling pathway suppressed hepatic cell proliferation	[59]
	70% PH		Mouse	Leucine-serine-lysine-leucine peptide promoted liver regeneration by the inhibition of TGF-β	[60]
**TGF-β**	2/3 PH		Mouse	BMP-9 (a member of the TGF-β family) disturbed the proliferative response and promoted fibrosis	[61]
		SFSLT (50%) 55–65 min cold ischemia	Rat	Administration of autologous adipose-derived mesenchymal stem cells increased IL-10 and TGF-β avoiding acute rejection and decreasing inflammatory responses	[62]
**TNFα**	2/3 PH		Mouse	Hepatocyte expression of ADAM17 (a major regulator of TNF, TNFR1, and AR amphiregulin) was not essential for hepatocyte proliferation in ADAM17 KO mice	[63]
	2/3 PH		Mouse	TNF-α injection exacerbates the regenerative failure in Gclm^−/−^ mice	[64]
		SFSLT (50%) 10 min or 10 h cold	Rat	TNFα expression was affected in a different way depending of the time of cold ischemia	[65]
**Adiponectin**	2/3 PH		Mouse	Adiponectin regulated regeneration controlling cell cycle progression, cytokine signaling and growth factor bioavailability	[66]
**Apelin**	70% PH		Mouse	The blockade of the apelin-APJ system pharmacologically by F13A promoted cell-cycle progression and liver regeneration	[67]
**Leptin**	70% PH		Rat	Leptin administration increased regeneration, liver weight and reduced damage	[68]
**Lipocalin**	2/3 PH		Mouse	LCN2 was induced in mice after PH although increased expression of LCN2 had no effects in hepatocyte proliferation	[24]
	2/3 PH		Mouse	In LCN2Hep^−/−^ after treatment with IL-6, hepatocyte-derived LCN2 promoted liver regeneration	[23]
	Major PH+I/R 20 min warm ischemia		Mouse: Steatotic and non-steatotic livers	Using wild type and mice over expressing LCN2, it was observed that LCN2 had deleterious effects in steatotic livers	[69]
	70% or 40% PH		Rat	Expression of the LCN2 mRNA was higher in 70% than in 40% PH	[70]
**Lipocalin**		SFSLT (55–70%). 40 min cold ischemia	Rat: Steatotic and non-steatotic livers	LCN2 is upregulated in steatotic small liver grafts. LCN2 exacerbated graft injury and promoted macrophage infiltration	[69]
**Vifastin**	70% PH 1 h warm ischemia		Rat: Steatotic and non-steatotic livers	Visfatin administration impaired damage and regenerative response in steatotic livers	[58]
**Angiotensinogen and Angiotensin**	70% PH		Mouse: Colorectal cancer liver metastases induction	Captopril (an inhibitor of renin–angiotensin system) did not impair liver regeneration Captopril exerted its effects on established tumors at only late stage acting as an angiogenic inhibitor, reducing widely tumor vessel density and enhancing tumor cell apoptosis	[71]
	70% PH		Mouse	Captopril enhanced early liver regeneration, effect associated with increased hepatic stem cells and MMP-9 protein	[72]
**HB-EGF**	2/3 PH		Mouse	Using focal adhesion kinase (FAK) KO mice, the authors show that Fakdeficiency enhanced liver regeneration modulating TNFα/HB-EGF axis	[73]
**HGF**	70% PH		Rat	Low-power laser irradiation enhanced the HGF/Met axis and Akt and Erk pathways improving liver regeneration	[74]
**IGF**	70% PH		Mouse	IGF-2 induced hepatocyte proliferation	[75]
**VEGF**	68% PH		Mouse	Hif2a-Vegf axed as a prime regulator of regenerative sinusoidal endothelial cells-hepatocyte crosstalk and revealed a crucial role for oxygen during liver regeneration	[76]
	90% PH		Rat: Steatotic and non-steatotic livers	Treatment with VEGF improved survival and stimulated liver regeneration	[77]
**VEGF**	90% PH		Rat	VEGF-sdf1 pathway in the liver is upregulated after PH, and this increases bone marrow production of progenitors of sinusoidal endothelial cells, which are required for liver regeneration	[78]
	70% PH		Rat	The over-expression of VEGF following surgery promoted angiogenesis	[79]
	70% PH		Rat	After transplant stem cells and MSCs transfected with VEGF, an increment in proliferation of hepatocytes was observed. VEGF transected MSCs also promoted the secretion of several growth factors as HGF and PDGF. These effects supported liver function and regeneration	[80]
	70% PH		Rat	In rats exposed to chemotherapy, the treatment with Bevacizumab (Anti-VEGF-A) did not affect liver cells proliferation after surgery	[81]
		SFSLT (50%) 55–65 min. cold ischemia	Rat	The over-expression of VEGF induced hepatocyte proliferation and neovascularization of the remnant liver	[82]

Ang: angiotensin; PH: partial hepatectomy; MM9-protein: Matrix metallopeptidase 9; min: minutes; I/R: ischemic reperfusion; HGF: hepatocyte growth factor; Akt: Protein kinase B; ErK: extracellular signal-regulated kinase; PH: partial hepatectomy; lipocalin: LCN; mRNA: messenger ribonucleic acid; VEGF: vascular endothelial growth factor; MSCs: mesenquimal stem cells; TGF-β: tumor growth factor-β; PDGF: platelet-derived growth factor; HB-EGF: heparin binding epidermal growth factor; FGF: fibroblast growth factor; BMP-9: bone morphogenetic protein 9; NO: nitric oxide; SFSLT: small-for-size liver transplantation; IL-6: interleukin 6; MCL-1: myeloid cell leukemia1; Bcl-2: B-cell lymphoma 2; JAK: JAK kinase; CREB: cAMP response-element-binding; PAI-1: phosphoribosylanthranilate isomerase 1; TNF-α: tumor necrosis factor alpha; Gclm: glutamate-cysteine ligase.

**Table 3:** Future perspectives in the use of adipokines implied in regeneration and/or liver diseases as therapeutic target to alleviate I/R injury and improve liver regeneration in the surgery of hepatic resections and partial liver transplantation.

Studies Reported in the Last Five Years			
Name	PH	Partial LT	Future Perspectives
**IL-6**	6	2	Studies performed using different % of PH. IL-6 could be a potential target to promote hepatocyte proliferation and decrease damage. However we must to be cautious because the controversial results in the presence of tumorigenesis and the fact that any of the authors evaluated its effect in PH under I/R
**PAI-1**	1	0	Only one reported study in an experimental mouse model of PH. PAI-1 was associated with a decrease in hepatocyte proliferation. More studies in the setting of PH under I/R, partial LT as well as considering steatotic status are required
**Resistin**	1	0	Its role in partial liver transplantation from steatotic and non-steatotic grafts has not been described. Further studies are required to consider its relevance
**TGF-β**	3	1	Deleterious effect in the hepatic proliferative response
**TNFα**	2	1	Although implied in hepatocyte proliferation, controversial results have been reported. Further studies are necessaries to elucidate the precise role of TNF-α in regeneration in surgical procedures as well as in the presence of steatosis
**Adiponectin**	1	0	As in the previous case, not many studies have been recently reported results and its modulation could beneficiate specially the outcome of hepatic resection in steatotic livers
**Apelin**	1	0	Only one study published make mandatory more research focused in this adipokine
**Leptin**	1	0	Not many works have been reported in the setting of PH, PH+I/R or partial LT. Since leptin deficiency impaired liver regeneration in obese mice, drugs aimed to modulate this adipokine would improve prognosis in liver transplantation from steatotic donors
**Lipocalin**	4	1	Controversial results even using the same experimental model of surgery. More studies are necessaries
**Vifastin**	1	0	Only one study published make mandatory more research focused in this adipokine
**Angiotensinogen and Angiotensin**	2	0	Promising results for cancer patients subjected to hepatic resection although more studies are necessaries
**HB-EGF**	1	0	Only one study published make mandatory more research focused in this adipokine
**HGF**	1	0	Only one study published make mandatory more research focused in this adipokine
**IGF**	1	0	Only one study published make mandatory more research focused in this adipokine
**VEGF**	6	1	Wide consensus in the published results even when different surgical procedures are compared. The benefits of VEGF for liver function and proliferation point that its pharmacological modulation would improve prognosis after surgery

LT: liver transplantation; Ang: angiotensin; I/R: ischemic reperfusion; HGF: hepatocyte growth factor; PH: partial hepatectomy; lipocalin: LCN; VEGF: vascular endothelial growth factor; TGF-β: tumor growth factor-β; HB-EGF: heparin binding epidermal growth factor; FGF: fibroblast growth factor; IL-6: interleukin 6; PAI-1: phosphoribosylanthranilate isomerase 1; TNF-α: tumor necrosis factor alpha; IGF: insulin growth factor.

## Abbreviations

LDLT	Living donor liver transplantation
I/R	Ischemia reperfusion
LT	Liver transplantation
SFS	Liver resection and small-for-size
SFSLT	Liver resection and small-for-size liver transplantation
LCN	Lipocalin
PH	Partial hepatectomy
KO	Knockout
NAFLD	Non-alcoholic fatty liver disease
NASH	Non-alcoholic steatohepatitis
EGF	Epidermal growth factor
Ang	Angiotensin
MM9-protein	Matrix metallopeptidase 9
min	Minutes
HGF	Hepatocyte growth factor
Akt	Protein kinase B
ErK	Extracellular signal–regulated kinase
mRNA	Messenger ribonucleic acid
VEGF	Vascular endothelial growth factor
MSCs	Mesenquimal stem cells
TGF-β	Tumor growth factor-β
PDGF	Platelet-derived growth factor
HB-EGF	Heparin binding epidermal growth factor
FGF	Fibroblast growth factor
BMP-9	Bone morphogenetic protein 9
NO	Nitric oxide
IL-6	Interleukin 6
MCL-1	Myeloid cell leukemia1
Bcl-2	B-cell lymphoma 2
JAK	JAK kinase
CREB	cAMP response-element-binding
PAI-1	Phosphoribosylanthranilateisomerase 1
TNF-α	Tumor necrosis factor alpha
Gclm	Glutamate-cysteine ligase
mRNA	Messenger ribonucleic acid
DKL-1	Delta like-1 homologue
IGF	Insulin growth factor
NGF	Nerve growth factor
HIF-2 α	Hypoxia-inducible factor alpha
NAD	Nicotinamide adenine dinucleotide
